# Primary Hyperoxaluria Type 2 Masquerading as Chronic Kidney Disease of Unknown Origin in an Adolescent: A Case Report

**DOI:** 10.7759/cureus.90466

**Published:** 2025-08-19

**Authors:** Muskan Garg, Sandeep Garg, Manidipa Mondal, Pujan Acharya, Lovish Garg

**Affiliations:** 1 Medicine, Maulana Azad Medical College and Associated Lok Nayak Hospital, Delhi, IND; 2 Orthopedics, Government Medical College & Hospital, Chandigarh, Chandigarh, IND

**Keywords:** combined liver kidney transplant, grhpr gene, phenotypic heterogeneity, primary hyperoxaluria type 2, young ckd

## Abstract

Primary hyperoxaluria (PH) is a rare autosomal recessive disorder of glyoxylate metabolism characterized by hepatic overproduction of oxalate, leading to oxalate nephropathy and systemic oxalosis without early intervention. PH1 contributes to 80% of the cases, while PH2 (10%) and PH3 (10%) being relatively rarer. PH carries a prevalence of one to three per one million population, and PH2 (pathogenic GRHPR gene variant) exhibits high phenotypic heterogeneity, complicating its diagnosis. We present a 17-year-old male who presented with a subacute onset of fever, dyspnea, oliguria, and volume overload for two weeks. Laboratory findings revealed: hemoglobin 5.2 g/dL, urea 194 mg/dL, creatinine 10.6 mg/dL, calcium 5.8 mg/dL, albumin 3.8 g/dL, and bicarbonate 12 mmol/L. Urinalysis showed 10-12 pus cells with significant proteinuria (2256 mg/day). Blood pressure was persistently elevated during hospitalization, and ultrasound revealed small kidneys (right 8.7 × 3.5 cm, left 8.3 × 4.1 cm). Evaluation for secondary causes of hypertension and chronic kidney disease (CKD) in the young patients was unremarkable; however, imaging studies identified nephrocalcinosis and urinalysis confirmed hyperoxaluria (96 mg/day) with elevated plasma oxalate levels (93 μmol/L). Secondary hyperoxaluria was excluded, and genetic analysis for PH confirmed a homozygous missense mutation (c.349T>C) in exon 4 of the GRHPR gene. High suspicion, accurate diagnosis, and timely interventions are mandatory to halt the progression of the disease in PH2. Management remains challenging, often necessitating intensive dialysis and consideration for either isolated kidney or combined liver-kidney transplantation as the definitive cure, with novel therapies such as RNA interference agents currently under investigation.

## Introduction

Primary hyperoxaluria (PH) is a rare autosomal recessive disorder of glyoxylate metabolism, leading to hepatic overproduction of nephrotoxic oxalate, which can lead to oxalate nephropathy and systemic oxalosis. It is classified into three types-PH type 1 (AGXT-alanine glyoxylate aminotransferase gene), PH type 2 (GRHPR-glyoxylate reductase/hydroxypyruvate reductase gene) and PH type 3 (HOGA 1-4-hydroxy-2-oxoglutarate aldolase 1 gene) with PH type 1 (80%) contributing to most of the cases, and type 2 (10%) and 3 (10%) being relatively rarer among them [1-3]. PH carries a prevalence of one to three per one million population in the US, with an approximate incidence rate of approximately one in 100,000 live births per year in Europe and one in 58,000 population worldwide; and PH2 prevalence was found to be seven times rarer than PH1 (carrier frequency: 1:279, prevalence, 1:310,055) [2-6]. Early diagnosis that requires high suspicion with metabolic and genetic workup and timely intervention is crucial to halt the progression of this life-threatening disease. A 17-year-old male with no significant history or risk factors presented to our hospital with features of end-stage renal disease (ESRD), who later turned out to be a case of PH type 2 oxalate nephropathy with a rare mutation. Besides the diagnostic challenges of PH type 2, it also necessitates management with intensive dialysis with either an isolated kidney or a combined kidney and liver transplant as the definitive treatment of this disorder.

## Case presentation

Here we are presenting a case of a 17-year-old male, resident of Delhi, belonging to poor socioeconomic strata, born of a consanguineous marriage, and the elder of the two siblings, who was referred to our hospital from a primary clinic for evaluation. The patient had been in his usual state of health until two weeks before the current presentation, when he developed fever, progressive shortness of breath (associated with orthopnea), and gradually developed bilateral pedal edema. He also had developed decreased urine output since last two to three days and had two episodes of generalized convulsive seizures since last one day. Fever was high-grade, intermittent, and associated with dry cough, chills, and burning micturition. There was no history of chest pain/palpitations/paroxysms of nocturnal dyspnoea/altered sensorium/vomiting/headache/blurry vision/any weakness in any limb or facial deviation/rash/abdominal pain or distension/haematuria/change in urine frequency or volume/frothy urine/facial puffiness/acute or chronic diarrhea/significant weight change/any swelling over body. There was no history of tuberculosis/tuberculosis exposure/any drug intake/complementary or alternative medicine uptake/blood transfusion. There was no significant family or perinatal history. There was no history of any similar complaints in the past. At the current presentation, pallor and mild symmetrical bilateral pitting edema were present. His temperature was 38.8°C, heart rate 110 beats per minute, blood pressure 170/100 mm Hg, respiratory rate 22 breaths per minute, BMI 22.4 kg/m^2^, jugular venous pressure (JVP) 6 cm of H_2_O above the right atrium, random blood sugar (RBS) 140 mg/dl, and the oxygen saturation 90% while the patient was breathing ambient air. Bilateral basal fine rales were present in both lungs. Fundus examination didn't show any features of retinopathy or any other abnormality. The rest of his systemic examination was unremarkable.

Initial investigations revealed a hemoglobin=5.2 g/dl, white-cell count=10,800 per μl, platelets=1,65,000 per μl, urea=194 mg/dl, creatinine=10.6 mg/dl (estimated glomerular filtration rate (eGFR)=6.5 mL/min/1.73 m²), Na=133 mmol/l, K=3.5 mmol/l, Mg=2.0 mmol/l, Ca=5.8 mg/dl, P=8.9 mg/dl, total bilirubin=1.0 mmol/l, aspartate aminotransferase (AST)=46 U/L, alanine aminotransferase (ALT)=16 U/L, alkaline phosphatase (ALP)=116 U/L, pH=7.12, HCO_3_=12 mmol/L, pCO_2_=28 mm Hg. His ECG had no significant abnormality, and urine analysis showed 10-12 pus cells, 3+ proteins, with no other abnormality. He was found to be hepatitis B positive; serology for hepatitis C and HIV was negative. CSF analysis was sent, which was acellular, with sugar 73 mg/dl (50-80 mg/dl), protein 41 mg/dl (15-45 mg/dl), and adenosine deaminase (ADA)=7 U/L (<10 U/L). In radiological studies, his chest x-ray showed bilateral pleural effusion, and the non-contrast computerized tomography (NCCT) head didn't reveal any abnormality. Ultrasonography of abdomen revealed-right kidney: 8.7 x 3.5 cm, left kidney: 8.3 x 4.1 cm and both kidneys had increased cortical echo with loss of cortico-medullary differentiation with multiple renal stones with largest stones measuring 1.3 x 1.1 cm and 1.2 x 0.9 cm in the left and right kidney respectively; liver, portal vein, common bile duct, pancreas and spleen were normal; mild ascites was present.

On the basis of the above-mentioned history, examination, and lab analysis, a provisional diagnosis of hepatitis B positive, young hypertensive with nephropathy, likely chronic kidney disease (CKD), who presented with urinary tract infection, which could be an aggravating factor responsible for acute decompensation; and generalized seizures likely secondary to hypocalcemia or hypertensive encephalopathy. He also had anemia, hypocalcemia, and small-sized kidneys; all pointing towards chronicity of the disease. Diagnostic workup for evaluation of hypertension and CKD in such a young patient was planned, and management decisions were made meanwhile.

As per institutional protocol, intravenous antibiotics were given, as were intravenous calcium, levetiracetam, furosemide, sevelamer, sodium bicarbonate, and carvedilol. Patient was transfused with packed red blood cells, and dialysis was commenced in view of severe resistant metabolic acidosis and volume overload. Patient's laboratory parameters were as shown in Table 1 during the first admission. 

**Table 1:** Laboratory parameters of the patient during the initial days of presentation. ALP: alkaline phosphatase, ALT: alanine aminotransferase, AST: aspartate aminotransferase; HCO₃: bicarbonate, mg/dl: milligrams per deciliter, mm Hg: millimeters of mercury (pressure unit), mmol/L: millimoles per liter, PCO₂: partial pressure of carbon dioxide, U/L: units per liter, μl: microliter.

Variable	Reference range for adults (in our hospital)	Day 1	Day 4	Day 7
Hemoglobin(g/dl)	13.2-16.6	5.2	7.8	8.2
White-cell count (per μl)	4500-11,000	10,800	9700	8060
Neutrophils	1800-7000	9072	6596	6045
Lymphocytes	3000-9500	972	2428	1450
Monocytes	200-1900	432	388	322
Eosinophils	50-450	324	291	241
Basophils	0-500	0	0	0
Platelets (per μl)	1,40,000-4,40,000	1,65,000	1,55,000	2,20,000
Urea (mg/dl)	19-43	194	172	146
Creatinine (mg/dl)	0.6-1.2	10.6	8.8	7.9
Sodium (mmol/l)	137-145	133	134	134
Potassium (mmol/l)	3.5-5.1	3.5	3.8	3.9
Calcium (mg/dl)	8.1-10.4	5.8	7.5	7.7
Phosphate (mg/dl)	2.5-4.5	8.9	8.8	7.9
Magnesium (mg/dl)	1.6-2.3	2.0	2.1	1.9
Total bilirubin (mg/dl)	0.2-1.3	1	0.9	0.8
AST (U/L)	17-59	46	54	51
ALT (U/L)	<50	16	43	27
ALP (U/L)	38-126	116	111	122
Serum protein (g/dl)	6.3-8.2	7	7.2	7.1
Serum albumin (g/dl)	3.5-5.0	3.8	3.9	3.7
pH	7.35-7.45	7.12	7.30	7.31
HCO_3_ (mmol/L)	22-26	12	19	20
PCO_2_ (mm Hg)	32-45	28	38	39

Patient's symptoms were gradually improving with no fresh episode of seizure. His metabolic profile also improved, including improvement in renal function, calcium levels, and increased pH; so he was managed with the same treatment protocol. Blood culture and urine culture were sterile. Other investigations, including evaluation for hypertension in young patients and CKD, are shown in Table 2.

**Table 2:** Serum, urine, and radiological investigations of the patient. 2D ECHO: two-dimensional echocardiography, ANA: antinuclear antibody, Anti-dsDNA: anti-double-stranded deoxyribonucleic acid, DCT: direct Coombs test, FT3: free triiodothyronine, FT4: free thyroxine, HbA1C: hemoglobin A1C, HBV: hepatitis B virus, HDL: high-density lipoprotein, LDH: lactate dehydrogenase, LDL: low-density lipoprotein, MCH: mean corpuscular hemoglobin, MCHC: mean corpuscular hemoglobin concentration, MCV: mean corpuscular volume, MRI: magnetic resonance imaging, PAC: plasma aldosterone concentration, PRA: plasma renin activity, PTH: parathyroid hormone, TC: total cholesterol, TG: triglycerides, TIBC: total iron-binding capacity, TSH: thyroid-stimulating hormone, UA: uric acid, WNL: within normal limits.

Investigation	Reference range (if applicable)	Results
Blood investigations		
Serum vitamin D (ng/ml)	30-100	13
Serum PTH levels (pg/ml)	13.6-85.8	112
HbA1C (%)	≤5.6	5.4
FT3 (pmol/L)	4.2-8.1	5.6
FT4 (pmol/L)	10-28	22
TSH (mIU/L)	0.4-4.04	3.3
TG (mg/dl)	40-149	111
TC (mg/dl)	80-200	165
HDL (mg/dl)	Low <40, high >60	55
LDL (mg/dl)	<100	79
LDH (U/L)	140-280	372
UA (mg/dl)	2.5–7.0	6.8
Serum iron (mcg/dL)	49-181	170
TIBC (mcg/dL)	265-497	181
Transferrin saturation (%)	20-50	18
Ferritin (ng/ml)	6.2-137	98
Vitamin B12 (pg/ml)	239-931	235
Folic acid (ng/ml)	2.7-20	10.4
MCV (fl)	80-100	97
MCHC (g/dL)	32-36	31
MCH (pg/cell)	27-31	29
Peripheral smear of blood	NA	Normocytic normochromic anemia, anisocytosis, few nucleated RBCs, few macrocytes, no schistocytes or any other atypical cells
DCT	NA	Negative
Homocysteine (micromol/L)	6.6-14.8	12
HBV DNA levels (IU/mL)	1.30-8.23 log	1.70 log
ANA	NA	Not detected
Anti ds DNA (IU/mL)	<10	Not detected
Serum cortisol (mcg/dL)	6-24	10
PRA (ng/mL/h)	1.2-2.4	1.8
PAC (ng/dL)	7-30	10.4
C3, C4	NA	WNL
Urine investigations		
Urine pH	4.5-8.0	6.5
Specific gravity	1.005–1.030	1.012
Urine culture	NA	Sterile
24 hours urinary protein	<150 mg/day	2256
Urine acid-fast bacilli	NA	Negative
Urine for active sediments	NA	No dysmorphic RBCs, no active sediments
24 h urinary metanephrines (mcg/24 h)	<300	86
Radiological investigations		
2D ECHO	NA	Normal study
Bilateral renal artery doppler and venous doppler	NA	Normal study
MRI brain	NA	Normal study

**Figure 1:**
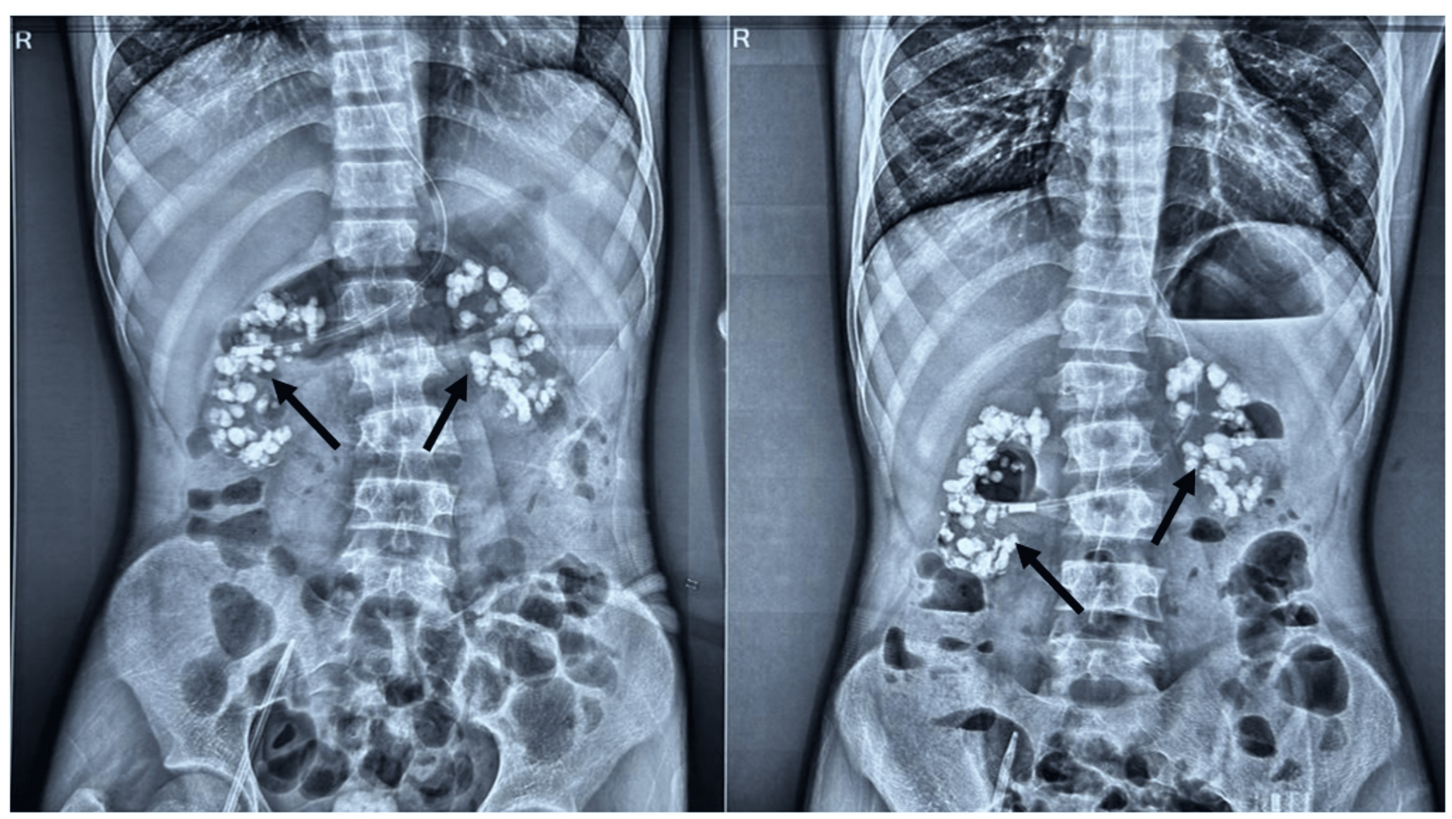
X-ray abdomen film of our patient showing bilateral kidney stones (marked by black arrows).

**Figure 2:**
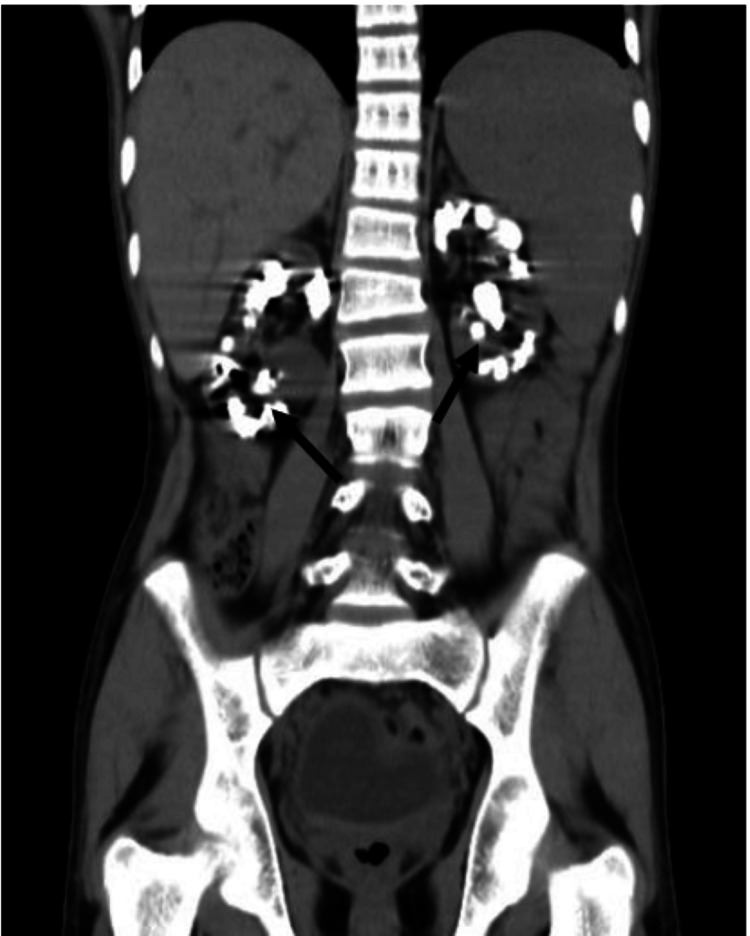
NCCT KUB film of our patient showing multiple large bilateral stones, suggesting bilateral nephrocalcinosis, affecting both cortex and medulla, with medullary predomination in coronal section (marked by black arrows). NCCT KUB: non-contrast computerized tomography of kidney, ureter, and bladder.

**Figure 3:**
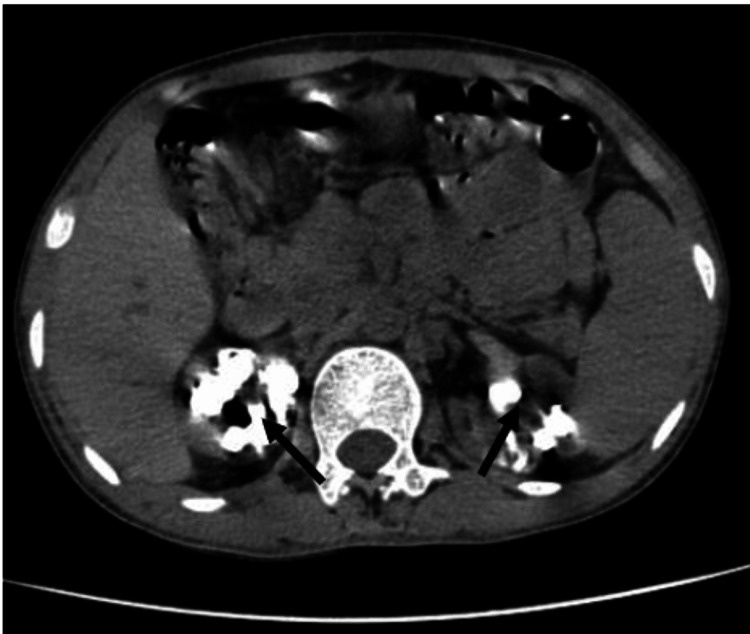
NCCT KUB film of our patient showing multiple large bilateral stones, suggesting bilateral nephrocalcinosis, affecting both cortex and medulla, with medullary predomination in transverse section (marked by black arrows). NCCT KUB: non-contrast computerized tomography of kidney, ureter, and bladder.

Repeat ultrasound of the kidneys was planned and showed bilateral shrunken kidneys with loss of cortico-medullary differentiation and multiple stones in both cortex and medulla, suggestive of nephrocalcinosis. An X-ray of the abdomen was done, which showed multiple large stones in the bilateral kidneys, as shown in Figure 1. Further, the patient underwent non-contrast computerized tomography of kidney, ureter, and bladder (NCCT KUB), which revealed predominantly medullary nephrocalcinosis in bilateral kidneys, shown in Figures 2, 3. All the above features and investigations indicated that nephrocalcinosis was the underlying cause behind the patient's landing with the advanced stages of CKD, and probably hypertension was secondary to it; we performed more investigations keeping causes of nephrocalcinosis in our mind.

Urine analysis was done, which revealed-urine calcium 5.5 mmol/24 h (2.7-7.5), urine phosphate 22 mmol/l (13-42), urine citrate 3 mmol/24 h (1.3-6), urine uric acid 6 mg/dl (4-8.5) and urine oxalate 96 mg/24 h (10-45) (confirmed twice) suggestive of hyperoxaluria. Plasma oxalate levels were 93 μmol/L (1-6). Urine L-glycerate levels were unavailable in our hospital.

There was no history of any increased intake of vitamin C or D supplements/nuts/tea/spinach/star fruit/chocolate intake, chronic diarrhea, or any other symptoms of inflammatory bowel disease; thus, ruling out secondary hyperoxaluria. Systemic oxalosis could also not be appreciated on 2D ECHO, X-ray skeletal survey, retinal examination, etc. Skin and vessels were not thickened; NCV was normal, and no periodontal disease was present. Genetic studies to rule out primary hyperoxaluria were sent simultaneously.

**Figure 4:**
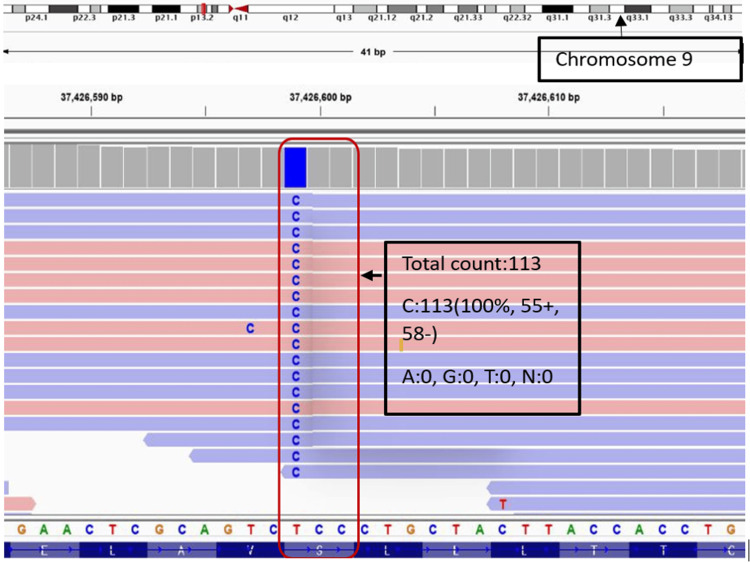
Genetic study showing mutation in the exon 4 of the GRHPR gene on chromosome 9 (chr9:g.37426599) showing substitution of proline for serine at codon 117 (method used: next-generation sequencing).

The patient was managed on the basis of suspicion of hyperoxaluria causing chronic kidney disease (CKD). Patient was started on tablet pyridoxine 5 mg/kg/day (augments activity of AGXT enzyme); coupled with orthophosphate (30 mg/kg), potassium citrate (0.15 g/kg), and magnesium oxide (400 mg/day) to suppress oxalate absorption and oxalate precipitation in urine. The patient was advised to have adequate fluid intake according to urine output; low-fat, sodium-restricted, normal calcium diet, and to decrease oxalate intake (spinach, soy products, nuts, potatoes, beet, navy beans, raspberries, dates, barley, etc.). As the patient's clinical and metabolic profile was improving with dialysis and the above-mentioned treatment, he was discharged on a hemodialysis regimen, to be followed with repeat urinary oxalate levels and genetic studies' results. Renal, liver, or bone marrow biopsies were not planned as he was symptomatically improving and reluctance of relatives. No stone analysis could be done as he didn't pass any stone, and no surgical intervention to remove stones was planned because of the advanced stage of disease and shrunken kidneys. He was counseled to undergo registration for kidney transplantation (or combined liver and kidney transplantation if required). Later, the patient was readmitted after 35 days with community-acquired pneumonia, uremic complications, and hypertensive posterior reversible encephalopathy syndrome, as the patient was non-compliant with the treatment. His repeat urinary oxalate levels were 88 mg/24h. Patient was again started on dialysis, and appropriate management was done according to standard treatment guidelines. His genetic analysis report (method: next generation sequencing) revealed: a very rare homozygous missense variant (c.349T>C) in exon 4 of the GRHPR gene on chromosome 9 (chr9:g.37426599T>C; depth: 112x) that results in the amino acid substitution of proline for serine at codon 117 (p. Ser117Pro; ENST00000318158.11) as shown in Figure 4, confirming the diagnosis of PH type 2 in our case; no pathogenic variant in AGT or HOGA1 gene was found. There was no history of recurrent stones or any acute or chronic renal failure in his family. His family was advised for genetic study, urinary and plasma oxalate measurement, radiographical studies of kidneys, but due to financial constraints, it could not be done. Patient deteriorated despite all the measures and succumbed to death in the second admission of 40 days.

Coincidentally, another homozygous autosomal recessive mutation with unknown significance was found in the MTR gene (c.2915A>G; p.Tyr972Cys, depth: 130X) that could result in homocystinuria-megaloblastic anemia. Though serum homocysteine levels (12 μmol/L) and vitamin B12 (235 pg/ml) were slightly deranged in our case, this mutation needs further validation.

Final diagnosis: 17-year-old male, hepatitis B positive, primary hyperoxaluria type 2 (homozygous missense variant (c.349T>C) in exon 4 of the GRHPR gene), causing nephrocalcinosis and ESRD.

## Discussion

PH type 2 is caused by a pathogenic GRHPR enzyme, predominantly found in the cytosol of hepatic cells, with its activity also identified in bone marrow cells, fibroblasts, and peripheral blood lymphocytes, thus questioning liver transplant as the definitive cure [7]. According to the CRIC study, there was an independent association between higher 24-hour urine oxalate excretion (even within the normal reference range) and 32% higher risk of CKD progression [8]. Oxalate was also linked with cardiac fibrosis and arterial hypertension in C57BL/6 mice, which needs to be verified in human studies [9]. PH type 2 possesses high clinical heterogeneity, which further impedes its diagnosis. It may manifest at any age, ranging from infantile nephrocalcinosis to recurrent kidney stones and progressive nephrocalcinosis in children and adults (80% cases), causing ESRD in approximately 20-30% of them; later ending up in systemic oxalosis when eGFR falls below ≤30-45 mL/min/1.73 m^2^ [5,10-13]. Oxalate deposition in the bone, kidneys, skin, retina, cardiovascular, and central nervous systems can occur in systemic oxalosis [2,11-13]. PH type 2, as reported previously, has a less severe phenotype than type 1, with average age of symptoms onset, diagnosis, and ESRD as 3.2 (1.0-11.3) years, 9 (2.0-32.0) years, 40 years (15.6-74.8), respectively [7]. However, in our case, this patient presented with ESRD directly at the age of 17 years without any prior episode of urolithiasis or nephrolithiasis in his infancy or childhood, thus pointing towards phenotypic heterogeneity of PH type 2 and similar or more severity than PH type 1 which has average age of ESRD diagnosis-24 years and 20-50% of them presenting with ESRD at the time of diagnosis [10]. Thirty-nine disease-causing mutations have been identified causing PH type 2 (Human Gene Mutation Database), and the variant reported in our case was found to be pathogenic according to the Global Variome shared LOVD database.

Management and challenges

PH possesses many challenges once ESRD develops, as there is a need for an intensive dialysis regimen, intermittent daily hemodialysis, with overnight peritoneal dialysis together constituting six to eight hours of dialysis daily with high flux dialyzers to achieve desired oxalate clearance with target plasma oxalate levels <30 μmol/L at the end of each dialysis session [14,15]. Furthermore, conventional hemodialysis and peritoneal dialysis have limited oxalate clearance (950-1440 μmol/day), lower than the daily oxalate production (3500-7500 μmol) in PH2 patients [14,15]. Moreover, systemic oxalosis contributes to difficulty in managing these patients as anemia in oxalosis is often erythropoietin (EPO) resistant because of bone marrow involvement; it is troublesome to make fistula because of oxalate deposition in skin and vessels, causing difficult vascular access, etc. It is also hypothesized that the higher oxalate in the urine, despite being in the reference range, might play a role in causing tubular atrophy, fibrosis, and acute kidney injury, ultimately leading to CKD; and whether treating this hyperoxaluria without nephrolithiasis can influence the progression of CKD in primary hyperoxaluric patients' needs to be confirmed [8,16,17]. Transplantation is the ultimate cure of the disease; however, dispute lies to decide between isolated kidney; or combined or sequential liver-kidney transplant. Even after transplantation, hyperoxaluria can still persist due to mobilization of tissue oxalate stores after the development of systemic oxalosis.

Novel approaches

Several therapies are under investigation to fight with PH and halt its progression to not land up in ESRD. It includes RNA interference therapeutic agents like nedosiran targeting hepatic lactate dehydrogenase and reducing conversion of glyoxylate to oxalate; and lumasiran [18,19]. Although both have been FDA approved for using in PH1, there is still limited evidence of their efficacy in PH2. Stiripentol, an inhibitor of LDH, may have a favorable effect in all types of PH [20]. Other experimental therapies include hepatocyte cell transplantation, gene-editing technology (clustered regularly interspaced short palindromic repeats) to correct specific enzyme pathways, etc. These novel approaches may potentially play a role in lessening the need of transplantation and mitigate the mortality in PH2 patients.

## Conclusions

This case highlights the diagnostic and therapeutic complexities of PH2, a rare autosomal recessive disorder with significant phenotypic variability. The patient's presentation with ESRD at 17 years, without prior nephrolithiasis, demonstrates the potential severity of PH2. High suspicion, early detection, and targeted management are critical to alleviate the nephrotoxicity of oxalate and prevent the development of ESRD and systemic complications. Comprehensive metabolic evaluation, genetic analysis, and tailored management, including potential liver and kidney transplantation, remain pivotal in improving outcomes in such cases. The report further contributes to the growing understanding of the variability and clinical severity of PH2, which may rival that of PH1 in certain cases.
